# Exteroceptive expectations modulate interoceptive processing: repetition-suppression effects for visual and heartbeat evoked potentials

**DOI:** 10.1038/s41598-017-16595-9

**Published:** 2017-11-28

**Authors:** Amanda C. Marshall, Antje Gentsch, Valentina Jelinčić, Simone Schütz-Bosbach

**Affiliations:** Department of Psychology, General and Experimental Psychology Unit, Ludwig-Maximilians University, D-80802 Munich, Germany

## Abstract

Interoception refers to the signaling of internal bodily commands. Here, we explore repetition suppression of intero- and exteroceptive neural markers to test whether the perception and predictability of exteroceptive stimulus material affects their expression. Participants completed a repetition suppression paradigm in which angry or neutral facial expressions repeated or alternated. Participants received either an implicit (experiment 1) or explicit (experiment 2) cue enabling the formation of expectations regarding the upcoming facial expression. We measured the heartbeat-evoked potential (HEP) indexing cardiac processing and visual evoked potentials (VEP) in response to viewing the second (repeated or alternated) face. Repeating angry facial expressions produced repetition suppression of both HEP and VEP amplitude while repeating neutral expressions led to repetition enhancement of HEP amplitude. This effect was magnified when participants were explicitly aware of predictive cues. Furthermore, repetition suppression of HEP amplitude correlated with neural attenuation of VEP activity. Results highlight repetition effects for interoceptive as well as exteroceptive neural markers and support top-down, expectation-based accounts of the phenomenon. Furthermore, results demonstrate that the perception of exteroceptive stimulus information has an effect on the processing of interoceptive signals and suggest a direct neural connection between the processing of external and internal sensory information.

## Introduction

Interoception refers to the processing (signaling) of homeostatic commands sent to monitor the body’s internal physiological state^1^. The largely subconscious processing of internal visceral signals is regarded as a fundamental basis for the construction of an embodied sense of self, promoting affect regulation and successful interaction with the external environment^2–5^. Recently, there has been a resurgence of scientific interest in interoception partly based on its wide-ranging implications for understanding and treating conditions such as Schizophrenia^6^ and depersonalization-derealisation disorder^7^ and its relevance for promoting immersive experiences in virtual reality settings used to treat chronic pain^8^. Existing studies have largely explored interoception by focussing on heartbeat perception^9^ as a means to quantify internal awareness. EEG studies exploring internal awareness have linked the signaling of cardiac signals to a specific ERP component manifesting with a fronto-central distribution around 200 to 400 ms following the R-wave in the electrocardiogram^10–13^. Increased amplitude of the heartbeat evoked potential (HEP) has been shown to coincide with increased heartrate detection accuracy^14,15^. Furthermore, source modelling of the component has indicated its origin in the right insula and anterior cingulate cortex^11^, brain structures strongly linked to interoceptive signaling^13^. To this effect, recent work by Park and colleagues^16^ demonstrated that experimentally manipulating participants’ sense of self-identification coincided with modulated HEP expression in the insula, thereby extending evidence for the component’s link to cognitive processes involving self-consciousness. Past work thus implicates HEP amplitude as a promising neural signal to measure the signaling of interoceptive commands in the form of cardiac activity. This has led to its use in studies exploring the way interoceptive signaling can affect the perception of external stimulus material. In this regard, Park and colleagues^13^ demonstrated that high amplitude of the HEP manifesting across the ventromedial prefrontal cortex and the inferior parietal lobule before the presentation of a faint visual stimulus predicted its conscious visual detection. Similarly focussing on the relationship between intero- and exteroceptive signaling, Salomon and colleagues^17^ reported reduced insula activity in response to a visual stimulus presented in synchrony with participants’ cardiac signal. Studies suggesting that interoceptive mechanisms can impact on our perception of the external environment highlight the importance of understanding the factors contributing to the processing of internal signals. However, work has yet to consider the reverse relationship by testing whether the perception of external stimuli likewise affects the processing of interoceptive signals.

A prominent phenomenon to study the neural signaling of sensory information is stimulus bound repetition suppression. Repetition suppression (RS) refers to the relative attenuation of a neural signal produced by the repeated occurrence of a stimulus^18^. RS has been extensively investigated for neural substrates reflecting the processing of external sensory signals^19^. In this capacity, it has been observed across different sensory modalities and various signatures of neural activity including single-cell recordings, electro- and magnetoencephalography and hemodynamic activity obtained via fMRI recordings^20^. Prominent theories have attributed RS to bottom-up processes resulting from the intake of sensory information into the cortex. Such accounts attribute RS to neuronal fatigue produced by the repeated firing of neurons tuned to a repeated stimulus^21^ or to a selective fine-tuning of neuronal assemblies processing the repeated stimulus in a more efficient manner^22,23^. More recent accounts introduce the idea of top-down expectations as the underlying mechanism enabling more efficient processing^23,24^. Here, repetition suppression is thought to reflect a reduction of prediction error (a neural signal evoked by the mismatch between an expected and observed state). Support for this top-down account of RS comes from studies demonstrating that valid expectations of a stimulus likewise produce reduced neural activity^25–27^ and that RS is most noticeable across highly predictable compared to volatile stimulus sequences^18,26^. For example, Summerfield and colleagues^28^ studied the attenuation of visual evoked potentials in a classical repetition suppression paradigm in which presentations of faces were either repeated or alternated. Importantly, they varied contexts which affected the predictability of repetitions, differentiating between high and low probability of a repeating stimulus and stable or volatile (rapidly changing) environments. The authors reported significantly decreased amplitude of a visual evoked potential manifesting across the vertex in response to viewing repeated faces. Crucially, they found that this effect was magnified by their manipulation of repetition probability. The authors interpreted these findings as support for a top-down account of RS, highlighting that contextual factors which affected repetition expectation significantly influenced their data pattern. However, other studies report RS effects in the absence of stimulus expectancy^29^. These seemingly conflicting patterns gave rise to the idea that bottom-up and top-down mechanisms may contribute to repetition suppression across different timescales^30,31^. Theories to this effect suggest that initial, low-level perception of local transition probabilities may evoke larger neuronal activity when a stimulus differs from its predecessor, thereby generating a bottom-up repetition suppression effect. At later timescales, higher-order expectations based on contextual statistical regularities may contribute to subsequent neural processing, thereby generating the effect in a top-down manner. This account was substantiated by the work of Todorovic and de Lange^20^ who were able to dissociate the impact of low-level perceptual and top-down expectation-based contributions for the suppression of early and late auditory evoked components respectively. Findings to this effect constitute a major development in understanding the underlying mechanisms governing RP effects and emphasise the advanced nature of research into RS produced by processing exteroceptive stimuli.

In this study, we explored repetition suppression in interoceptive systems by measuring HEP amplitude in response to viewing pairs of repeating or alternating stimuli chosen to evoke different patterns of cardiac activity. The aims of this study were threefold. Based on findings that interoceptive signaling affects exteroceptive perception, we aimed to explore whether the perception of exteroceptive stimulus material likewise affected internal states. To address the potential relationship between extero- and interoceptive processing, we additionally measured the amplitude of visual evoked potentials (VEP) elicited by viewing the second (repeated or alternated) stimulus. This allowed us to capture the standard repetition suppression effect established for processing visual information and explore a potential neural link signifying an interaction between the signaling of external and internal information. Secondly, we were able to explore repetition suppression for the signaling of intero- as well as exteroceptive signals which has remained a largely unexplored field. Thirdly, we presented stimuli in predictable and unpredictable contexts, a manipulation that allowed us to consider possible contrasting top-down and bottom-up accounts of repetition suppression and explore top-down contributions to interoception as has been suggested by recent theoretical models^1,32^.

Based on Summerfield and colleagues’ original design^28^, we thus implemented a probabilistic cue manipulation within a repetition suppression paradigm. The stimuli used in the paradigm consisted of two facial expressions. Exteroceptive viewing of emotional facial expressions has been found to modulate interoceptive signals. To this effect, past work has captured autonomic arousal in the form of elevated skin conductance and pupil dilation in response to viewing faces expressing different emotions, with the biggest difference between autonomic markers emerging consistently for the comparison between angry and neutral expressions^33,34^. Regarding cardiac activity, Jönsson and colleagues^35^ reported that men exhibited increased power in the high frequency heart rate power spectrum as well as decreased cardiac midinterval acceleration in response to angry relative to happy faces. Similarly, in women a single dose of testosterone has been shown to significantly accelerate cardiac responses to angry relative to neutral faces^36^. In addition to eliciting a markedly different expression of autonomic markers, past work has linked processing of angry faces to the orbitofrontal and anterior cingulate cortex, regions likewise implicated in interoceptive processes^37^. Based on this, we selected angry and neutral facial expressions to maximise the differential impact their presentation would have on cardiac activity. Thus, repeating expressions were designed to evoke highly similar patterns of cardiac activity across both stimulus iterations, thereby producing a repetition suppression effect for a repeating interoceptive signal. Conversely, alternating trials were conceived to produce dissimilar patterns of cardiac activity, necessitating a re-processing of novel cardiac signals without neural attenuation.

We varied the predictable context in which the stimuli were presented by introducing a cue across two different types of blocks. In cued blocks, the fixation cross separating the first from the second face turned red whenever a repeated facial expression was about to occur, thus predicting repetitions with 100% certainty. In uncued blocks, the fixation cross turned red equally often for repeated and alternated trials and thus had no relationship to the trial sequence. This design also allowed us to implement elements of all contextual factors used in Summerfield’s paradigm by manipulating both expectation (high certainty of a repeated face in cued blocks) and volatility (stable environment due to absolute predictive certainty in cued blocks). We tested the impact of this cue across two experiments in which we explored whether its effectiveness depended on implicit (experiment 1) or explicit (experiment 2) cue knowledge.

In both the interoceptive and exteroceptive domain, we expect effects of stimulus repetition, that is an alteration of HEP and VEP amplitude, in repeated relative to alternated trials. In line with reports of increased repetition effects to salient, negative stimuli^38,39^, we expect this effect to be stronger for angry facial repetitions. If a top-down, expectation-based account of repetition suppression holds true, we further expect this effect to be magnified in cued relative to uncued blocks, in which the contextual cue enables the generation of reliable expectations imbued with a high degree of certainty.

## Materials and Methods

### Experiment 1

#### Participants

Twenty-five participants (10 female, all right-handed, mean age: 25.3 ± 3.9 years) with normal or corrected-to-normal vision took part in the study. All participants provided written informed consent and received payment or student credit for their participation. All procedures were approved by the ethics committee of the Ludwig-Maximilians University Munich (Ethikkommission der Fakultät für Psychologie und Pädagogik der Ludwig-Maximilians-Universität München) in accordance with the Declaration of Helsinki (BMF 1991; 302; 1194) and all methods used in this study were performed in accordance with the declaration’s guidelines and regulations. Depressive symptoms were assessed using the Beck Depression Inventory (BDI-II^40^). Current and general levels of anxiety were assessed using the State-Trait Anxiety Inventory (STAI^41^). Both symptoms may affect interoceptive signaling^42^. We thus included this measure to screen for any individuals scoring above the clinical cut-off point. However, all participants scored within the normal range. Required sample size was determined before data acquisition. A power analysis indicated we had 80% power to detect the small to medium effect (Cohen’s δ = 0.45; α = 0.05) of stimulus repetition on HEP amplitude observed in a previous pilot study.

#### Procedure

Before the main experiment, participants completed a heartbeat tracking task^43^. After a short training session (15 s), participants were instructed to report the number of heartbeats they silently counted during three fixed time intervals (25, 35, 45 s in randomised order). Instructions given to participants stated “Please concentrate on your own heartbeat for the duration in which a fixation cross is shown in the middle of the screen. Look at the cross and count the number of beats you can actually feel. Do not take your pulse or otherwise measure your heartbeat while doing this. Please report only felt heartbeats. Do not estimate or guess at a potential number, even if this means you report only few heartbeats.” With these stringent instructions, we explicitly discouraged participants estimating or guessing the number of heartbeats^44^. Heartbeat perception score was calculated using the following formula:

$$\frac{1}{3}\sum (1-[(recorded\, heartbeats-counted\, heartbeats)\,\div \,divrecorded\,heartbeats])$$

**Figure 1:**
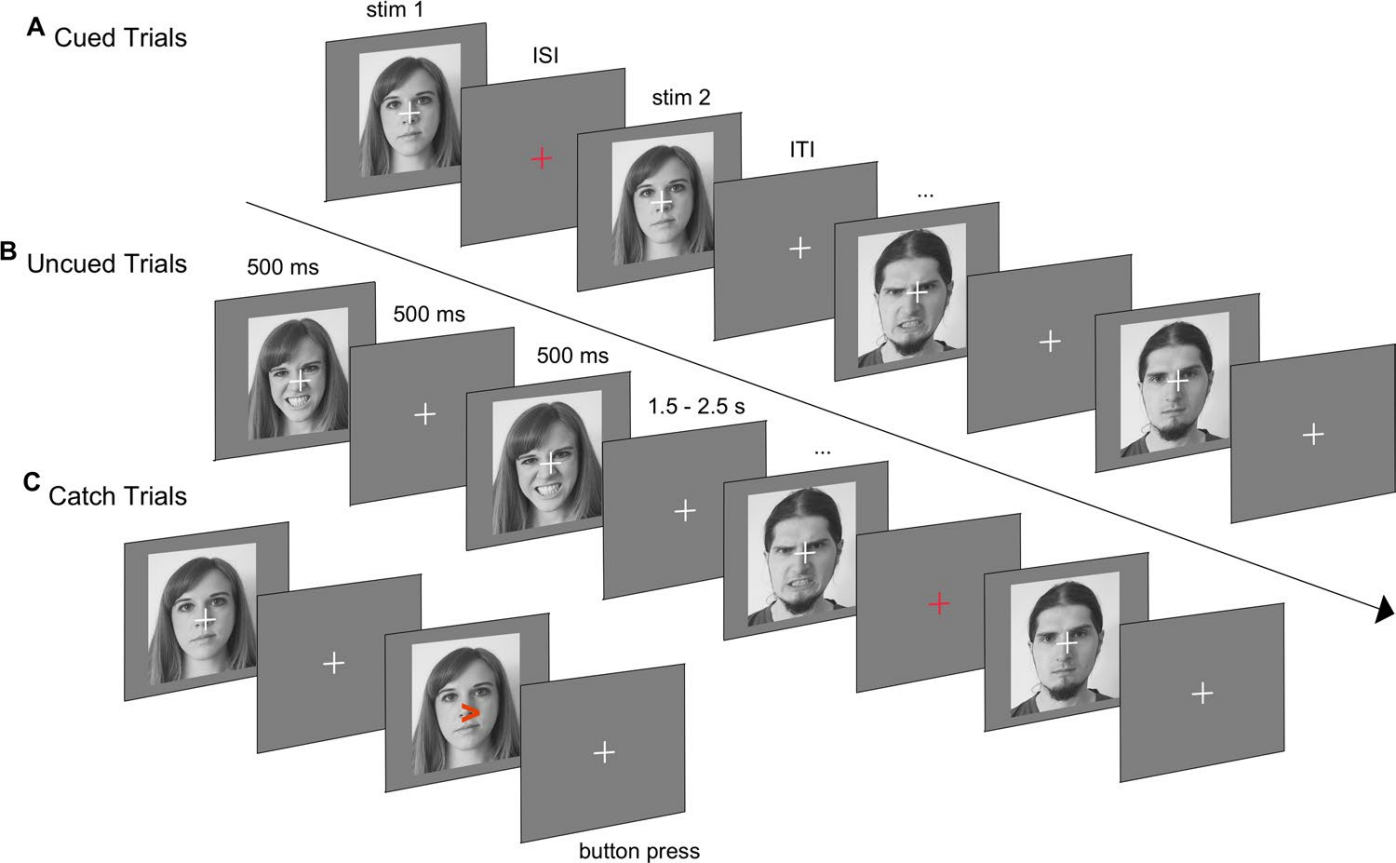
Repetition suppression paradigm. Face stimuli were presented in successive pairs interspersed by a blank screen. A further blank screen of ∼2 s separated trials. (**A**) An example of cued trials. The fixation cross in the inter-stimulus interval turns red if the same facial expression is repeated and remains white for alternating expressions. (**B**) An example of uncued trials. The fixation cross in the inter-stimulus interval turns red equally often for repeated and alternated facial expressions. (**C**) Example catch trial. Participants were required to press a button corresponding to the direction the arrow pointed in. Arrows appeared on 20% of all trials. These trials were excluded from later analyses. Faces presented in this figure were self-generated for the purpose of distribution under a creative commons license. Actual faces used in the study were sourced from the validated NimStim set of facial expressions.

The main experimental session consisted of a repetition-suppression paradigm^30^ (see Fig. 1). Stimuli for the paradigm were taken from a validated set of face stimuli (NimStim^45^) and included 40 young actors (20 females, 20 males) modelling a neutral and angry facial expression. Each of the 80 stimuli was used for only one trial per block. After a brief training session (15 trials), participants completed a total of 14 experimental blocks. Each block consisted of 40 trials. In each trial, the same face was presented twice in succession for a duration of 500 ms, interspersed with a 500 ms fixation screen. A jittered inter-trial interval ranging from 1.5–2.5 seconds separated the current from the successive trial. The presented face either wore the same facial expression across both presentations (repetition trials) or differed so that a neutral facial expression was followed by an angry expression or vice versa (alternation trials). Trials were presented in two different stimulus contexts. In cued blocks, the fixation cross separating the first from the second face turned red whenever a repetition of the same expression occurred (20 trials) and remained white for an alternation (20 trials). In uncued blocks, the colour change of the fixation cross was split equally between alternation and repetition trials (10 trials each). Thus, in uncued blocks, the colour change of the fixation cross was unrelated to stimulus sequence and could not be used as a rule to predict the upcoming facial expression. Participants were not informed of the purpose behind the appearance of the red fixation cross. To enable participants to discern the purpose, we presented cued and uncued blocks as a unit, counter-balanced across participants. Thus, half the participants completed the cued blocks before moving on to the uncued blocks while the other half of participants encountered the opposite order. Participants’ task was to monitor the stimulus sequence for occasional arrows pointing to the left or right. Arrows were superimposed on the first or second face, and participants were asked to respond to their appearance by pressing a left or right response button for which they received immediate feedback. These catch trials served to ensure participants’ continued attention and occurred on 20% of all trials (equally often for repetitions/alternations and on the first or second face). Catch trials were discarded from later analyses. The experimental session ended after participants filled out the questionnaires. For experiment 1, participants were asked to provide a short answer indicating whether they had noticed any particular pattern in the stimulus sequence.

#### EEG recording and processing

Continuous EEG signals were collected using a 64-channel active electrode system (actiCAP, Brain products GmbH, Gilching, Germany) at a sampling rate of 500 Hz. Bipolar ECG electrodes were placed below the left clavicle and below the left pectoral muscle.

Offline EEG data were pre-processed using EEGLAB (EEGLAB 9.0.3, University of San Diego, San Diego, CA) and BrainVision Analyzer (BrainVision Analyzer 2.0, Brain products GmbH). In EEGLAB, continuous EEG data were filtered between 1 and 40 Hz and re-referenced to a common average reference. Independent component analysis was conducted on the continuous EEG signals and stereotypical components reflecting eye movements, blinks and the cardiac field artefact (CFA) were removed based on the visual inspection of 40 independent components^15,46,47^. When exploring event related components locked to heartbeats, the cardiac field artefact (produced by a strong electric dipole generated by movement of the heart muscle) manifests as a strong electrical signal which can overlap with HEP amplitude and thereby influence results. A meaningful analysis of HEP expression therefore necessitates its removal. Components relating to the CFA as well as eye movements and blinks were characterized by a time course (projection matrix) and a scalp map (weighting matrix). We identified ICs relating to the CFA by searching for components displaying the bimodal topography commonly associated with this artefact^48^. In addition, we searched for a frequency peak around 5 Hz and a rhythmically repeating signal time course. The CFA tends to be expressed across two components with similar but slightly rotated scalp topographies. Thus, we removed 2–4 components per participants (average of 1.75 related to the CFA across participants). HEPs were computed on EEG signals locked to the R-peak of the ECG. R-peaks were detected using the EEGLAB plugin FMRIB 1.21^49^. In BrainVision Analyser, data for the visual ERPs reported by Summerfield and colleagues was segmented into −100 to 500 ms epochs relative to the onset of the second face. For the HEP, data was segmented into 1000 ms periods relative to the onset of the inter-trial interval marker. The minimal duration of the inter-trial interval lasted for 1.5 s. This manner of epoching therefore excluded potential overlap with subsequent trials. Within this post-stimulus interval, epochs were further segmented into periods ranging from −100 to 600 ms relative to the R-peak marker. Visual ERPs and HEPs were calculated by averaging across trials for each experimental condition using the −100 ms interval prior to stimulus onset or R-peak marker for baseline correction. In accordance with previous approaches^11,15^ a current-source-density (CSD) transformation was subsequently applied to HEP epochs to reduce potentially remaining contamination of HEPs by residual CFA overlap.

#### Statistical analysis

There is still a paucity of literature describing the topography and time course of the HEP. Furthermore, Summerfield and colleagues^28^ opted for a data-driven approach to determine how ERPs varied in response to stimulus repetition. We therefore chose a non-parametric, permutation-based approach to determine ERP morphology. We began by identifying neural phenomena that varied with the main effect of repetition (i.e. contrasting repetition and alternation trials) and calculated point-estimate statistics (F-values associated with the main effect of repetition) across the entire time window for each component. For the HEP, this time window covered the post-stimulus period after the second face had disappeared from the screen and ranged from −100 to 600 ms from the onset of the R-peak marker. For the VEP reported by Summerfield and colleagues, the time period ranged from −100 to 500 ms, covering the entire duration in which participants viewed the second face. Time windows were averaged into seven 100 ms windows (i.e. 100 ms ∼ 200 ms etc.) prior to the permutation analysis. We then permuted the dataset by shuffling across conditions and subjects and re-computing the statistics 1000 times, thus providing a null distribution corresponding to each time point. Across each permutation, the maximum F-value across the entire time window was logged, providing a distribution of maximal values obtained under the null hypothesis. We then compared original point-estimates to this distribution, choosing the values that fell into or above the 95^th^ percentile as significant candidates for subsequent analysis. This procedure provides exact statistics corrected for multiple comparisons and is a common approach used to study large neuroscientific datasets^28,50,51^. We determined ERP topography in the same manner. For this analysis, no electrode clusters were formed (i.e. all 64 electrodes were treated as a distinct variable). However, for the subsequent analysis, we collapsed across electrode clusters revealing a main effect of repetition to limit the number of comparisons. Thus, averaged ERP amplitudes from electrode pools and latencies exhibiting a main effect of repetition were submitted to a 2 (block: cued vs. uncued) × 2 (trial: repetition vs. alternation) × 2 (valence: negative vs. neutral) repeated measures ANOVA. Paired t-tests were used for follow-up analysis and in these cases Bonferroni-corrected p-values are reported. In addition to the standard frequentist approach, we reported Bayes Factors for all primary analyses. Bayesian Repeated Measures ANOVAs were conducted in JASP^52^ adopting the default prior settings (fixed effects = 0.5; random effects = 1; covariates = 0.354; auto sampling).

#### Control variables

In line with recent work exploring neural events linked to heartbeats^53,54^, surrogate R-peaks were created to explore whether observed HEP modulations were time-locked to cardiac events. We created a total of 400 surrogate R-peaks per participant which preserved the same interbeat interval and variability as the original by randomly shifting the onset of the original R-peak within a time window of −500 – +500 ms. R-peaks were shifted by the same amount separately for each subject and each of the eight conditions. We subsequently applied the same criteria for calculating HEP amplitude and submitted these surrogate values to the permutation and analysis of variance calculation.

In addition, we obtained HEP amplitudes and cardiac parameters from the recording interval of the heartbeat tracking task (average duration 204 s per participant). HEP amplitudes were quantified in a time window of 200–300 ms using the same approach previously described. Using the open source R-HRV package implemented in R^55^ we extracted the interbeat-interval as well as the heart period power spectrum at low (0.04–0.15 Hz) and high frequencies (0.15–0.40 Hz) for each participant. While low frequency power has been linked to sympathetic nervous system activity, parasympathetic activity is associated with the higher frequency range. To examine whether trait differences in any of these measures within our participant sample may have affected our HEP results, we performed an additional regression analysis in which we explored their impact on HEP amplitude manifesting during the heartbeat tracking interval.

### Results

#### Behavioural and questionnaire data

Participants responded accurately to 75.1 ± 13.3% of catch trials. Reaction times on response trials (mean = 427 ± 73 ms) did not differ as a function of any of our experimental manipulations (all p_s_ > 0.36; *BF*_10_ = *0.96*). Thus, the classical frequentist approach found no evidence that participants exhibited different levels of attention or vigilance across the experimental conditions while Bayes factors remained inconclusive. In answer to whether they had noticed a particular pattern associated with the red fixation cross only 16% of participants were able to explicitly identify the rule, suggesting that the cue remained at a subliminal level.

Participants’ questionnaire results (BDI = 7.6 ± 9.5; STAI trait = 37.1 ± 11.1; STAI state 37.6 ± 10.3) make the current sample comparable to previously reported student samples^56^. However, mean heartbeat tracking scores (0.51 ± 0.36) are lower than commonly reported and more comparable to those reported in depressed population samples^57^. We attributed this to the stringent instructions we gave to our participants. This resulted in four individuals who reported very low values for at least one of the three intervals. Recalculating accuracy scores without these outliers resulted in a score of 0.61 ± 0.31 which approximates previously reported scores. However, recalculating subsequent electrophysiological analysis without these outliers did not affect the results.

#### VEP response to second face

We observed a main effect of repetition manifesting between 100 and 200 ms post-stimulus. This effect was significant over two electrode clusters, situated across the vertex (Cz, CPz, CP1; Cohen’s δ > 0.2) and across parietal electrodes (P1, POz, PO3; Cohen’s δ > 0.25). No further main effects or interactions manifested for the mean voltage averaged across central electrodes and Bayes factors for these effects offered substantial evidence for H_0_ (all p_s_ > 0.05; *BF*_10_ = *0.15*). For the mean voltage averaged across parietal electrodes, we observed a significant block x trial interaction F(1,24) = 4.96, p = 0.036 (*BF*_10_ = *4.39*). However, no significant comparisons emerged at post-hoc level (all p_s_ > 0.05). Recalculating the analysis without the four individuals who explicitly identified the implicit cue’s purpose did not significantly change the results.

#### Heartbeat evoked potential

**Figure 2:**
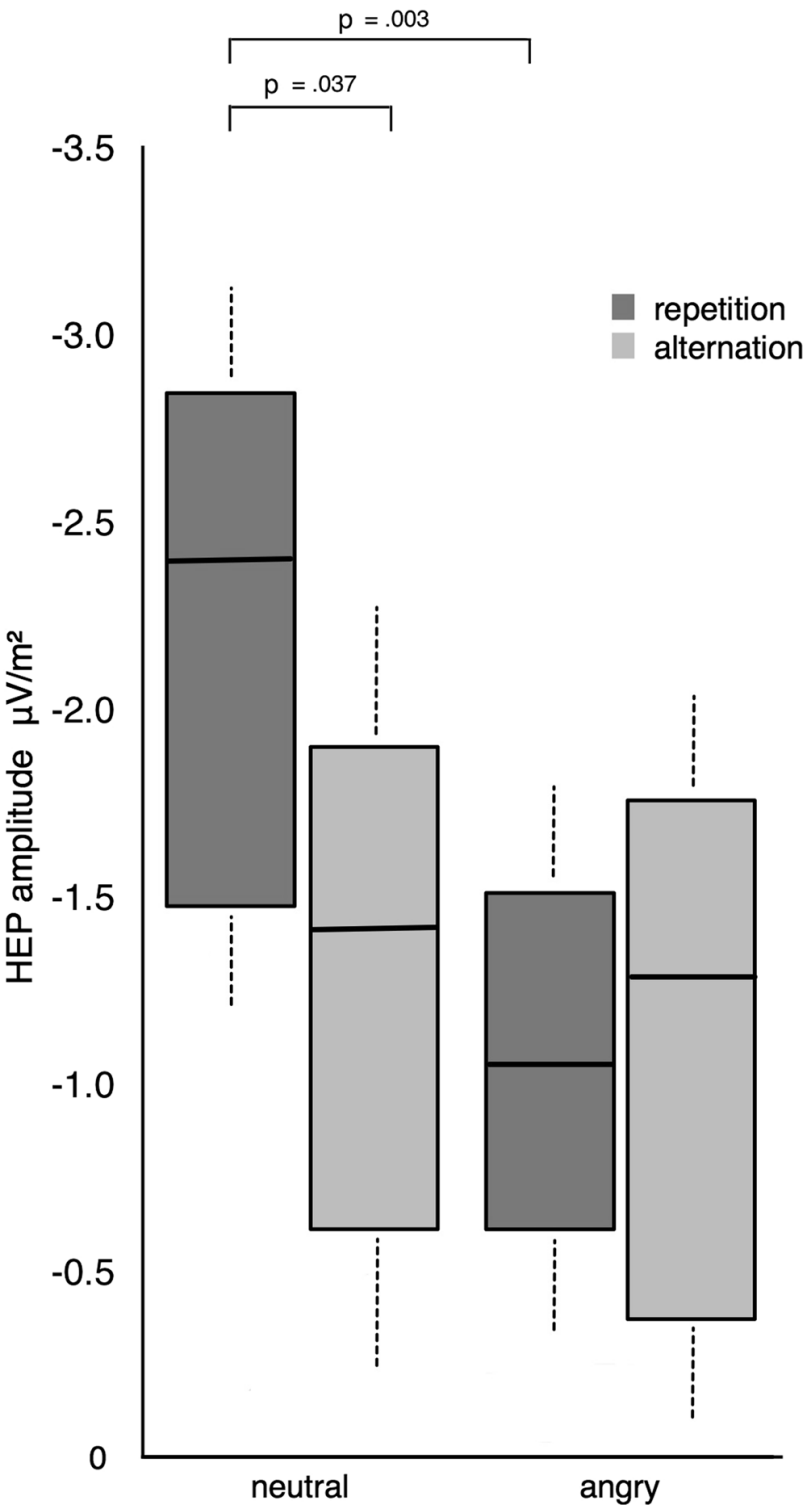
Box plots displaying the amplitude of HEPs (200–300 ms) for the trial by valence interaction observed for experiment 1 (whiskers represent standard deviations). Repeating neutral trials produced an elevation of HEP amplitude while repeating negative trials decreased HEP expression.

For the HEP, a main effect of repetition manifested between 200 and 300 ms over frontal-central electrodes (F1, Fz, F2, FC1, FCz, FC2; Cohen’s δ > 0.4), thus corresponding to previous descriptions of the HEP component^9,10^. In addition to the main effect of trial, analysis of the mean voltage computed across all significant electrode sites revealed a valence x trial interaction F(1,24) = 5.3, p = 0.03 (*BF*_10_ = *93.78*). Follow-up comparisons revealed a significantly higher HEP amplitude for repeated neutral (*Mean* = *−2.75*) compared to alternated neutral (*Mean* = *−1.89*) trials (p = 0.037), as well as a significantly enhanced HEP amplitude to repeated neutral (*Mean* = *−2.75*), relative to repeated angry (*Mean* = *−1.61*) trials (p = 0.003; see Fig. 2). Results thus reveal repetition enhancement of repeating neutral stimuli while indicating the opposing pattern (repetition suppression) for repeated negative stimuli (however this amplitude reduction did not reach significance when compared to angry alternated trials).

We further investigated whether HEP effects were related to participants’ explicit interoceptive accuracy as measured using the heartbeat perception task. We found no evidence to this effect; heartbeat perception scores did not correlate with absolute HEP amplitude collapsed across all conditions (**ρ** = −0.18, p = 0.4; *BF*_10_ = *0.11*). This corresponds to previous reports^13^ and indicates that HEP modulation reflects a transient online state rather than persistent offline interoceptive accuracy. Similarly to the VEP analysis, re-running the analysis without the individuals who explicitly identified the cue did not significantly alter the findings.

#### Control analysis

We performed extensive control analyses to test whether differential cardiac activity across experimental conditions could have confounded the results or produced spurious findings unrelated to an enhanced or modulated interoceptive response. To this effect, we extracted peak amplitudes of the ECG R-peak and mean activity of the ECG T-wave from ECG electrodes across the 200 to 300 ms time window in which we observed the HEP effect. Comparisons between experimental conditions did not reveal a differential modulation of the HEP by T-wave latency range (all p_s_ > 0.48). Similarly, Bayes factors found strong evidence for the absence of a difference of cardiac activity between experimental conditions *BF*_10_ = *0.047*). In addition, we extracted peak amplitudes from ECG and frontal-central electrodes (uncorrected HEP waveforms) during the 0 to 80 ms interval following R-wave onset. ECG electrodes showed no significant difference of heartrate expression to any of the experimental conditions and Bayes factors found substantial evidence for the absence of an effect (all p_s_ > 0.08; *BF*_10_ = *0.12*). Similarly, uncorrected HEP waveforms across frontal-central electrodes showed no differences in R-wave deflection across experimental conditions. Thus, data indicate that our effect manifested independently of cardiac influence as R and T-peak amplitudes across the ECG electrodes did not differ between conditions. In addition to exploring whether cardiac activity across experimental conditions affected HEP results, we also tested whether general variation in cardiac parameters across participants impacted on HEP expression. For this, we regressed the interbeat interval as well as high and low heart period power on HEP amplitude during the heartbeat tracking recording. The resulting model was non-significant F(3,23) = 1.11, p = 0.368, R^2^ = 0.14 (Bayes factors showed substantial evidence for H_0_: *BF*_10_ = *0.031)*. This indicated that general variability of cardiac measures did not significantly impact on HEP amplitude in our participant sample.

Importantly, our paradigm rests on the assumption that neutral and angry faces produce distinct patterns of cardiac activity. To test this assumption, we once more extracted peak amplitudes and latency of the ECG R-peak and mean activity of the ECG T-wave from ECG electrodes across the 500 ms time window in which participants observed the first facial expression. We observed reduced R-peak latencies t(24) = 2.14, p = 0.042 (Bayes factors showed substantial evidence for H_A_: *BF*_10_ = *4.57)* and increased mean T-wave activity (t(24) = 2.06, p = 0.43 (Bayes factors showed substantial evidence for H_A_: *BF*_10_ = *4.11)* for angry relative to neutral faces. Crucially, while this strengthens the assumption that face stimuli evoked distinct cardiac patterns, these effects were not observed during the time window in which we measured HEP amplitude. Thus, they are unlikely to have directly impacted HEP expression.

To examine whether removal of the cardiac field artefact affected HEP results, we recomputed the analysis across the frontal-central electrode pool for the 200–300 ms time window using waveforms uncorrected for the CFA. Results revealed the previously reported trial x valence interaction F(1,24) = 4.98, p = 0.041 (*BF*_10_ = *88.77* offered very strong support for H_A_). Thus, while partial overlap of the CFA with the HEP measured in the subsequent time window slightly reduces the interaction effect, we can rule out that CFA removal produced the observed effect.

To determine whether observed effects were truly locked to heartbeats, we re-ran first the permutation test to determine maximal HEP amplitude expression (400 iterations) using the previously created surrogate R-peaks. Comparing the resulting distribution of maxima (F-value point estimates) to the original for both the temporal and topographical analysis did not find a maxima distribution equal to or greater than that obtained using true R-peaks, thus indicating that randomly shifting the R-peaks in time negated the HEP effect comparing repeated to alternated trials. Similarly, we submitted values obtained from surrogate R-peaks over the same time period and electrode pool as values obtained with true R-peaks to the subsequent analysis of variance calculation. Resulting values found no significant main effects or interactions (all p_s_ > 0.8) and Bayes factors offered strong evidence for H_0_ (*BF*_10_ = *0.038*). Combined, results confirm that HEP expression and its subsequent modulation across stimulus conditions reflects cardiac activity rather than other changes in ongoing EEG activity such as for example HEP effects produced by viewing the second face.

In addition to the surrogate R-peak analysis, we specifically tested whether HEP results may be affected by neural processing related to the previous face stimuli. To this effect, we computed ERPs time locked to the appearance of the second face for the 200–300 ms time window during the inter trial interval (in which we report HEP effects) and submitted them to the same analysis as our HEP waveforms. We found no significant main effects or interactions relating to repetition or valence (all p_s_ > 0.22) and Bayes factors offered strong evidence for H_0_ (*BF*_10_ = *0.41*), which indicates that HEP findings were not influenced by temporal overlap with the visual ERP related to processing the second facial stimulus.

### Experiment 2

Results for experiment 1 found an impact of stimulus repetition on a trial level. While repeating angry facial expressions led to a reduction of HEP amplitude, repeated neutral expressions produced an increase in HEP amplitude. However, we did not observe an impact of our implicit cue manipulation. In experiment 2 we thus made the cue explicit rather than presenting it implicitly. This distinction allowed us to address the level of processing necessary for the cue to become effective. Within the framework of associative learning (i.e. matching the cue to a certain stimulus pattern in our design), the distinction between subliminal and conscious processing of stimuli is an important one. Past work in this respect has emphasised that associative learning depends on conscious cognitive processes that enable the formation of propositional knowledge^58^. Conversely, a number of learning theories postulate that associative links between stimulus sequences are either formed automatically or via an interplay between conscious and subconscious processes^59^. Past work exploring the interplay between interoceptive and exteroceptive perception has demonstrated that presenting exteroceptive stimuli in synchrony with subliminally processed internal bodily signals affects their conscious perception^13,17^. In exploring the reverse relationship, we thus wished to investigate at which processing level exteroceptive predictive cues exerted an impact on the expression of interoceptive (i.e. HEP) and exteroceptive (i.e. VEP) markers.

#### Participants

We recruited twenty-five participants (9 female, all right-handed, mean age: 26.7 ± 4.3 years) with normal or corrected-to-normal vision. We excluded one participant because their behavioural performance fell below the pre-determined cut-off (<75% explicit rule recognition). A previous power analysis indicated we had 80% power to detect the medium effect (Cohen’s δ = 0.5; α = 0.05) of stimulus repetition on HEP discovered in experiment 1.

#### Procedure

We followed the same procedure reported for experiment 1. After completing the heartbeat tracking task, participants moved on to the main experiment. This differed from the previous paradigm in two ways. Firstly, we pseudo-randomised the appearance of cued and uncued blocks. Secondly, participants were informed of the existence and nature of the red fixation cross. They were told that in 7 of the 14 blocks the fixation cross turning red signified a repeat of the same facial expression, while the cross remaining white signalled an alternation. Conversely, in the other half of blocks, the cross turned red randomly and did not convey any information about the upcoming stimulus. In addition to responding to catch trials, participants were told to monitor the behaviour of the fixation cross and indicate at the end of each block whether the cross had acted as a valid cue or not.

#### EEG processing and statistical analysis

We employed the same EEG/ECG set up, data pre-processing and statistical approach to determine ERP latencies and topographies reported for experiment 1.

### Data availability

Both datasets generated in the current study are available from the corresponding author on reasonable request.

### Results

#### Behavioural and questionnaire data

Participants successfully differentiated between cued and uncued blocks for 13.58 of 14 blocks (97%). In addition, they responded accurately to 71.2 ± 9.2% of catch trials (mean reaction time 479 ± 55 ms). An independent-samples t-test revealed that this performance did not significantly differ from that in experiment 1 (p > 0.05). Similarly Bayes factors for this difference offered substantial evidence for the absence of an effect (*BF*_10_ = *0.27*). This suggests that the two tasks of monitoring the rule and responding to catch trials did not significantly interfere with each other. Participants mean heartbeat perception score (0.53 ± 0.3), as well as their scores on the STAI (state: 37.9 ± 9.5; trait: 38.6 ± 9.9) and BDI (8.2 ± 7.9) compared to scores from participants tested in experiment 1.

#### VEP response to second face

**Figure 3:**
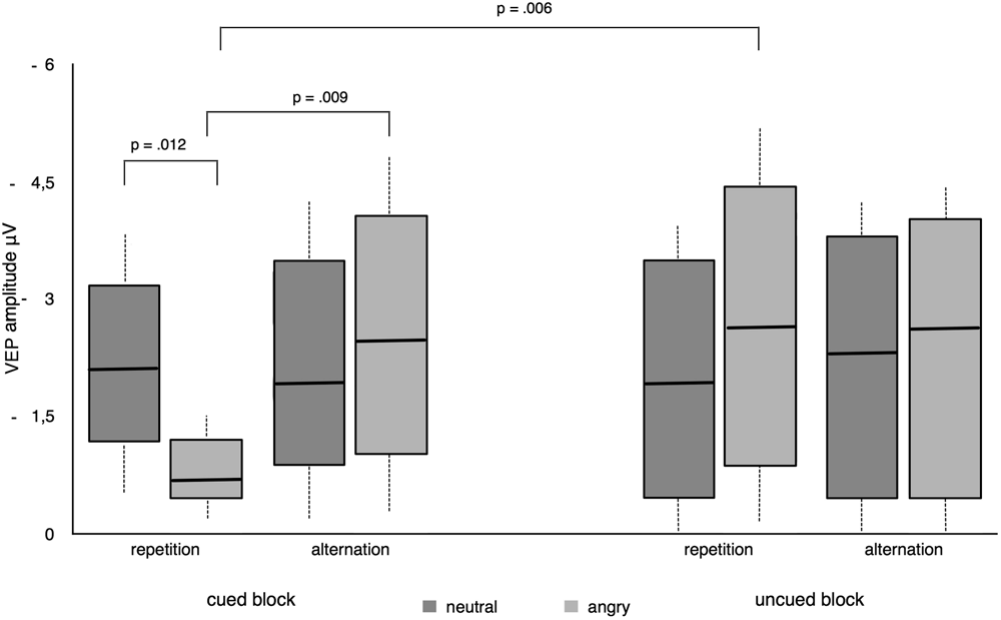
Box plots displaying the amplitudes of ERPs in response to viewing the second facial expression (whiskers represent standard deviations) for the interaction between block, trial and valence in experiment 2. During explicitly cued blocks, we observed a significant repetition suppression effect for repeated, angry facial expressions.

Once again, we observed a main effect of repetition 100–200 ms post-stimulus onset. This effect was weakly expressed over the vertex (Cz, C2, CPz; δ > 0.1) but reached maximal significance across parietal-occipital electrodes (PO3, PO5, POz, Oz, O1, CB1; δ > 0.5). For the mean amplitude averaged across parietal-occipital electrodes, we observed significant interactions between block x valence F(1,23) = 5.23, p = 0.028 (*BF*_10_ = *81.44* offered very strong evidence for H_A_) as well as block x trial F(1,23) = 5.76, p = 0.024 (*BF*_10_ = *122.74* offered very strong evidence for H_A_). Crucially, we also observed a significant 3-way interaction between block x trial x valence F(1,23) = 7.28, p = 0.013. Calculating the Bayes factor for this interaction likewise offered very strong support for the presence of an effect (*BF*_10_ = *96.11*). Exploring this interaction with follow-up tests indicated a significantly reduced ERP response to angry repeated faces in the cued block (*Mean* = *−1.43*) which significantly differed from repeated neutral faces in the cued block (*Mean* = *−3.87;* p = 0.012) and alternated angry faces in the cued block (*Mean* = *−4.37;* p = 0.009). In addition, decreased ERP amplitude to angry repeated faces in the cued block also significantly differed from angry repeated faces in the uncued block (*Mean* = *−4.53;* p = 0.006; see Fig. 3).

**Figure 4:**
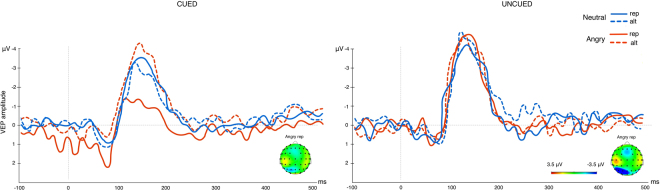
ERP amplitudes in response to viewing the second facial expression during explicitly cued and uncued blocks (experiment 2). Solid lines represent repeated facial expressions while dashed lines represent alternated facial expression trials. Topographies are displayed for VEP expression to the repeated angry face where the significant VEP modulation occurred.

This interaction persisted when re-running the analysis with the difference scores (subtracting 1^st^ from 2^nd^ face) F(1,23) = 7.33, p = 0.013 (*BF*_10_ = *93.98* offered strong support for H_A_). Observing the difference scores indicates that this interaction seems primarily driven by a high initial neural response to the first angry face in cued blocks (*Mean* = *−5.2*) which is greatly reduced for the second angry face in cued blocks (*Mean* = *− 1.4*). We did not observe this strong repetition suppression effect across any of the other stimulus conditions (see Fig. 4 for a breakdown of ERP expression across blocks in experiment 2).

#### Heartbeat evoked potential

**Figure 5:**
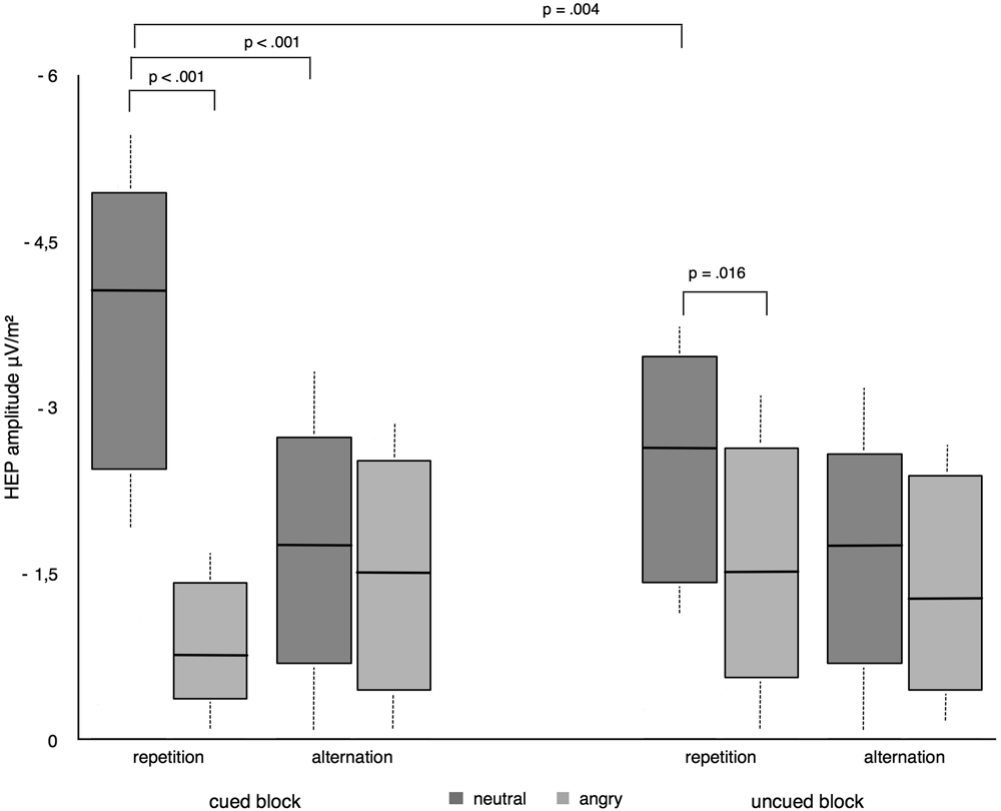
Box plots displaying the amplitudes of the HEP (200–300 ms) for the interaction between valence, trial and block in experiment 2 (whiskers represent standard deviations). HEP amplitude was elevated for repeated neutral trials and reduced in response to repeated angry trials. This effect was exacerbated in cued relative to uncued blocks.

**Figure 6:**
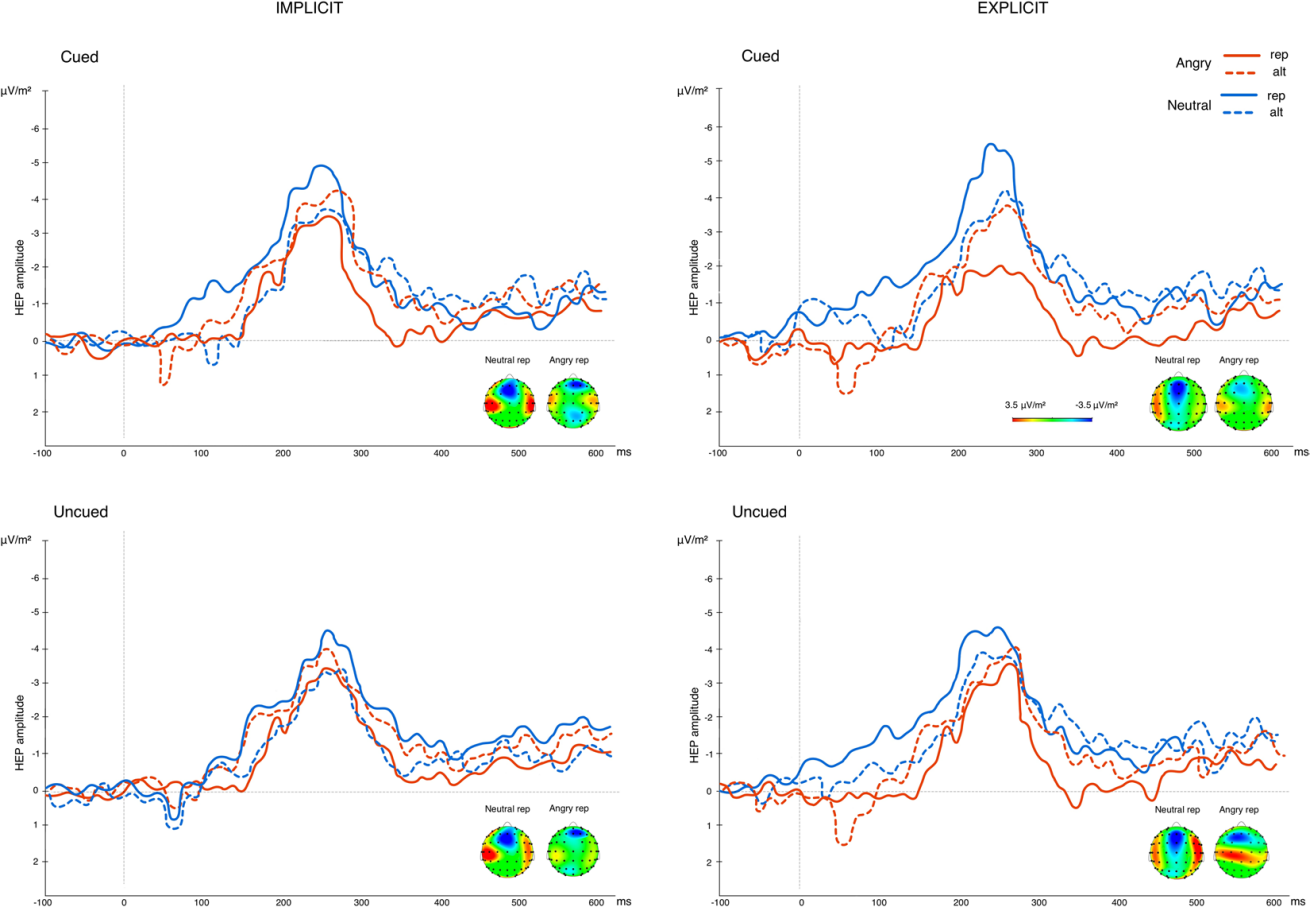
R-wave-locked HEP waveforms averaged across the fronto-central electrode pool (F1, Fz, F2, FC1, FCz, FC2). Waveforms are displayed across cued and uncued trials for the implicit cue (experiment 1) and the explicit cue (experiment 2). Solid lines represent repetition trials while dashed lines reflect alternation trials. Topographies are displayed for the modulation of the HEP in repeated angry and neutral trials across conditions.

We found a dominant main effect of repetition across the same 200–300 ms time window and the same frontal-central electrodes (F1, Fz, F2, FC1, FCz, FC2; Cohen’s δ > 0.4) observed in experiment 1. Analysis of the mean amplitude collapsed across electrode sites revealed a significant valence x trial interaction F(1,23) = 11.5, p = 0.003 (*BF*_10_ = *85.97* offered very strong evidence for H_A_), a significant valence x block interaction F(1,23) = 8.12, p = 0.009 (*BF*_10_ = 10*5.12*) as well as a significant 3-way interaction between block x trial x valence F(1,23) = 8.88, p = 0.007 (*BF*_*10*_ = *112.78*, offered very strong evidence for H_A_) (see Fig. 5). Follow-up comparisons of this interaction revealed a strong repetition enhancement of HEP amplitude for neutral repeated trials in the cued block (*Mean* = *−5.1*), accompanied by a strong repetition suppression effect for repeated angry faces in the cued block (*Mean* = *−1.9)*. This HEP depression in response to repeated negative faces differed significantly from HEP elevation to repeated neutral faces (p < 0.001), from HEP expression to alternated angry faces (*Mean* = *−3.2; p* < *0.003)* and from HEP expression to repeated angry faces in the uncued block (*Mean* = *−3.7; p* < *0.002)*. Similarly, increased HEP amplitude to repeated neutral trials in the cued block significantly differed from alternated neutral trials in the cued block (*Mean* = *−2.89;* p < 0.001) and neutral repeated trials in the uncued block (*Mean* = *−3.76*; p = 0.004). In addition, we observed a significant difference between neutral repeated (*Mean* = *−3.56*) and angry repeated faces (*Mean* = *−2.1;* p = 0.016) in the uncued block (see Fig. 6 for a comparison of HEP waveforms across conditions for both experiments). Findings from experiment 2 thus produce a repetition suppression effect for the repeated negative stimuli while replicating the repetition enhancement to repeating neutral stimuli observed in experiment 1.

**Figure 7:**
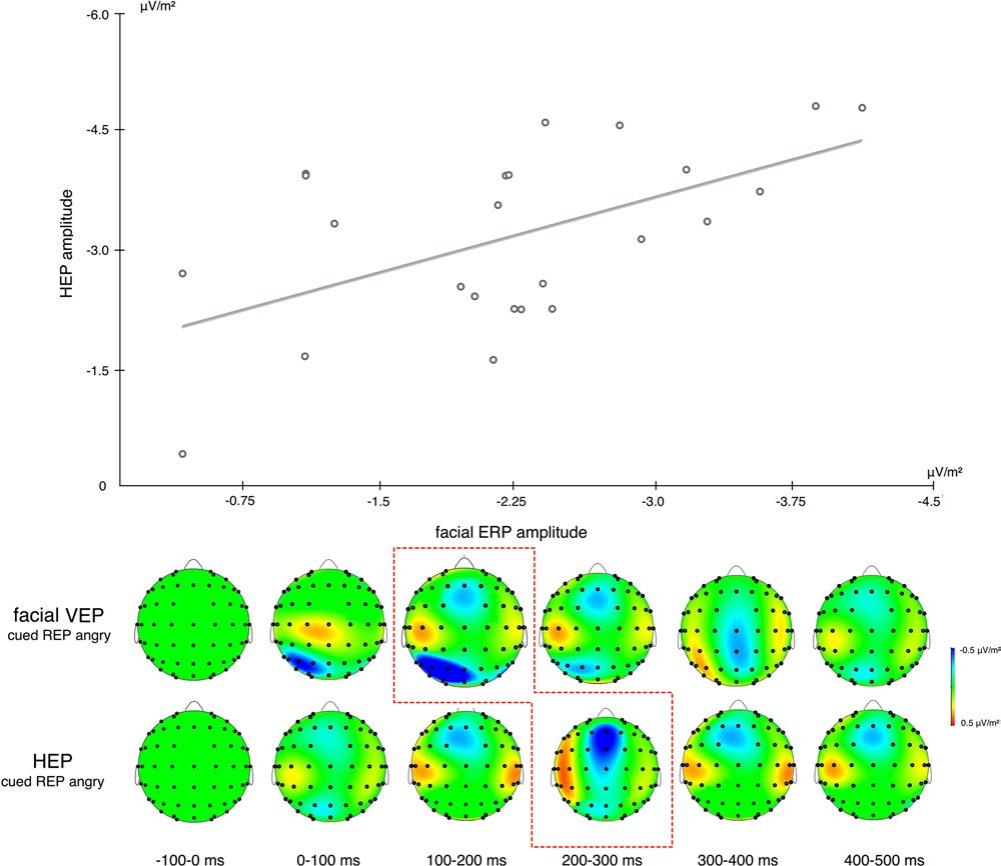
Topographies showing the time course of the facial ERP and the HEP during repeated trials of angry facial expressions. Maximum amplitude of the facial ERP manifests 100–200 ms after the appearance of the second face stimulus. Expression of the HEP reaches maximal amplitude at 200–300 ms in the subsequent (1.5 s) time window after the face has disappeared. The above scatter plot shows the strength of the correlation between both components.

Similar to experiment 1, the correlation between absolute HEP amplitude (collapsed across all conditions) and heartbeat tracking score failed to reach significance (**ρ** = −0.09, p = 0.7). Bayes factors for this correlation remained inconclusive (*BF*_10_ = *1.12*). Crucially, however we observed a significant correlation between the enhanced repetition suppression effect in the visual ERP response to repeated negative faces during cued facial processing blocks and the decreased HEP we observed for the same stimulus category during the subsequent time window (**ρ** = 0.48, p = 0.014; *BF*_10_ = *84.73* offers very strong evidence for H_A_ *;* see Fig. 7). This finding suggests an association between the neural signature to exteroceptive stimuli and the subsequent expression of interoceptive markers.

#### Control analysis

We undertook the same control analyses reported for experiment 1. T-wave latency did not differ between any of our experimental conditions (all p_s_ > 0.36; *BF*_10_ = *0.12* offers substantial evidence for H_0_). Thus, our HEP results manifest independently of differential expression of the heartrate across conditions. Similarly, peak amplitudes extracted 0–80 ms post R-wave from ECG and uncorrected frontal-central electrodes did not differ significantly between experimental conditions, although ECG amplitudes showed a trend towards a main effect of valence F(1,23) = 3.17, p = 0.092 (*BF*_10_ = *2.2* provides anecdotal evidence for H_A_) (all other p_s_ > 0.27; *BF*_10_ < *0.14* offering substantial evidence for H_0_). Similar to experiment 1, we found reduced R-peak latencies t(23) = 3.42, p = 0.032 (Bayes factors showed substantial evidence for H_A_: *BF*_10_ = *6.77)* and increased mean T-wave activity (t(23) = 2.85, p = 0.37 (Bayes factors showed substantial evidence for H_A_: *BF*_10_ = *5.71)* in response to viewing angry relative to neutral faces during the first stimulus presentation. Thus, faces once more elicited different patterns of cardiac activity while this effect no longer seemed present during later intervals of HEP measurement. Analysing HEP waveforms uncorrected for the cardiac field artefact revealed the same 3-way interaction between block, trial and valence F(1,23) = 4.27, p = 0.052 (*BF*_10_ = *72.93* offers very strong evidence for H_A_). Thus, while partial overlap of the CFA compromised the significance of the interaction, we can rule out that removal of the artefact produced the observed effect. Exploring the impact of the interbeat-interval and high and low frequency heart period power on the HEP manifesting during the heartrate tracking task resulted in a non-significant regression model F(3,22) = 1.76, p = 0.339, R^2^ = 0.16 (*BF*_10_ = *0.036* offers substantial evidence for H_0_). This indicates that variation in cardiac activity across participants did not affect the expression of HEP amplitude. Re-computing the permutation test (400 iterations) for the main effect of repetition in temporal and topographical domains with HEP amplitudes obtained from surrogate R-peaks did not yield a distribution of maximal F-values greater than or equal to that obtained using true R-peaks. Similarly, conducting the ANOVA with HEP values obtained using surrogate R-peaks using the same time window and electrode pool as the original analysis did not produce any significant results (p > 1.9, *BF*_10_ = *0.051* offers strong support for H_0_). Akin to experiment 1, results thus indicate that HEP expression was truly locked to the heartbeat. Finally, ERPs related to facial processing during the 200–300 ms time window in which we observed the HEP showed no significant main effects or interactions relating to late processing of facial components (all p_s_ > 0.44; *BF*_10_ < *0.14* provided substantial evidence for H_0_).

## Discussion

In this study, we explored how the perception of repeated or alternated exteroceptive stimuli affected HEP and VEP expression as an indicator of interoceptive cardiac signaling and exteroceptive visual perception respectively. To contrast bottom-up and top-down accounts of repetition suppression, we manipulated participants’ expectations of the stimulus sequence by introducing a cue that enabled more confident predictions about the upcoming stimulus sequence. This cue was presented implicitly (experiment 1) and explicitly (experiment 2). Relative to alternated trials, we report repetition enhancement of HEP amplitude for repeated neutral and repetition suppression of HEP amplitude for repeated angry faces. This effect was significantly magnified by the presence of an explicit cue. The presence of an explicit cue further produced a repetition suppression effect for VEP expression in response to repeated angry faces relative to alternated angry faces. Specifically, for repeated angry faces reduced VEP and HEP amplitude were found to significantly correlate.

Across both experiments, we observed strong effects of stimulus repetition. However, repetition suppression of the VEP and HEP manifested only in response to angry facial expressions in explicitly cued blocks. Conversely, the strongest repetition effect was enhancement of HEP amplitude to neutral repeated facial expressions, a pattern that manifested across conditions in both experiments. Repetition enhancement has been reported by several studies, often alongside repetition suppression effects^60^. Interestingly, reports attribute distinct temporal characteristics to both effects, with repetition suppression manifesting at early timescales (95–150 ms) while repetition enhancement occurs later (230–270 ms)^61^. Dissociations between both repetition effects have also been observed for different types of stimuli. For example, repetition suppression of the fMRI bold response has been reported for familiar faces and highly visible stimuli while unfamiliar faces and visually degraded displays produced repetition enhancement^62,63^, an effect which has been explained by the presence of pre-existing stimulus representations^62^.

More recent work emphasizes predictive learning as a core functional mechanism that may explain temporally dissociable repetition phenomena. In an fMRI study, Müller and colleagues^64^ observed that repetition effects for low contrast visual scenes followed an inverted U-curve. The first five stimulus presentations elicited gradually increasing repetition enhancement which changed to repetition suppression for further repetitions. This pattern suggests that repetition enhancement may be reflective of a learning mechanism in which the repetition of a stimulus leads to a gradual increase of perceptual predictions made at higher levels of the neuronal hierarchy. Repetition enhancement is usually observed at late latencies^65^. A top-down account of the effect therefore ties in with work emphasising the contribution of top-down, expectation-based mechanisms to repetition effects manifesting at later timescales^20^. After successfully establishing a perceptual representation of the stimulus, this precision control can be implemented at lower hierarchical levels, leading to suppression effects observed at early latencies. According to this account, repetition suppression can thus be understood as a more efficient mode of processing incoming stimuli achieved by the perceptual learning process indexed by repetition enhancement. This echoes recent theories of repetition suppression attributing neural attenuation to a progressive top-down optimisation of model complexity (reduction of prediction error) and provides a fitting explanation for the repetition suppression effect of the early onset VEP (100–200 ms) we observed in the current dataset. It further accounts for the enhancement we observed of the HEP component to neutral faces occurring at later timescales (200–300 ms). Increased HEP amplitude to repeating neutral faces could thus be tentatively interpreted as a reflection of interoceptive learning established by increasing familiarity to similar heartbeat patterns evoked by repeated facial expressions. This interpretation is consistent with work linking higher HEP amplitude to enhanced processing of cardiac signals^14–16^. However, it should be noted at this point that we did not replicate the link between HEP elevation and conscious awareness of interoceptive signals in the form of heartbeats. Across both experiments, HEP amplitude did not correlate with heartbeat tracking score. A potential explanation for this is that our HEP measurement is not equivalent to those reported in previous studies^14–16^. Typically, such correlations have been observed for HEP amplitudes obtained under conditions of rest. In contrast, HEP expression in our study was modulated by manipulations of exteroceptive stimuli. It thus differs from previous reports in which HEP amplitude is measured without this modulating influence. This qualitative difference in the manner of HEP expression may have influenced an effect which has been highlighted as hard to replicate based on its low statistical power^66^ and its susceptibility to confounding factors such as the ability to accurately judge time intervals^67,68^. Moreover, past work has shown that an increase of HEP amplitude with interoceptive learning may take place unconsciously^69^. To establish whether the HEP acts as a valid maker for conscious interoceptive signalling, future work should thus systematically explore the impact of contextual factors on HEP characteristics. Further evidence for an interoceptive learning account for the observed HEP elevation comes from a recent ERP study showing HEP enhancement as a function of cardiac interoceptive learning^69^. The study in question trained participants to follow their own heartbeat using auditory feedback and observed higher HEP amplitude in those participants who accurately learned to tap in synchrony with their own cardiac signals.

A top-down interpretation of HEP enhancement and VEP suppression is supported by finding both effects magnified by the cue manipulation. Cued blocks enabled participants to form accurate expectations about the upcoming stimulus sequence, thus leading to a successful approximation of repeating cardiac and visual signals. This process may have enhanced interoceptive learning (indexed by higher HEP amplitude to neutral faces) and subsequent model updating to minimise prediction error (indexed by reduced VEP amplitude in explicitly cued blocks). Our observation that repetition effects are modulated by contextual cues corresponds to past work of Summerfield and colleagues^28^ as well as Todorovic *et al*.^20^, both of whom demonstrated that manipulations which enhance participants’ expectations about upcoming perceptual information produce stronger repetition suppression effects of early visual and auditory evoked ERPs. Further support for a top-down account of the repetition effects observed for the HEP lies in the time course at which this component manifested. Generally, event related potentials occurring at short latencies are classified as reflecting low-level perceptual processes, while later ERP components are thought to reflect higher order operations such as associative learning or model updating^70^. Thus, the late latency of the HEP, sometimes manifesting up to 600 ms after the R-peak in the ECG^69^ lends further credence to a top-down mechanism governing its expression. The current dataset therefore lends support to a growing body of work^1,32^ implicating a top-down generation of repetition suppression effects while indicating that top-down learning mechanisms, indexed by repetition enhancement, may govern the signaling of interoceptive commands. Interestingly, the effect of our global cue manipulation only manifested once this was made explicit. This suggests that only conscious certainty that expectations about an upcoming stimulus are correct generates a perceptual learning and model updating effect which is strong enough to be captured by extero- and interoceptive neural markers. Our findings in this regard thus correspond to theories of associative learning which highlight the necessity of conscious cognitive processes to form propositional knowledge which is then used to make stimulus associations^58^.

However, this interpretation does not account for the attenuation of HEP amplitude produced in response to repeating angry faces. We observed a strong valence effect across both experiments which was likewise amplified by the introduction of an explicit cue in experiment 2. As mentioned above, the HEP is a late component. HEP repetition suppression to repeating negative faces is thus unlikely to reflect the same mechanism producing repetition suppression of the VEP, that is a more fine-tuned signaling of cardiac signals at low perceptual levels due to a minimisation of prediction error (which is implemented by higher levels of the sensory hierarchy).

A possible explanation may lie in considering the contributing factor of attention. Attention has been shown to modulate repetition suppression, producing stronger neural attenuation for attended relative to ignored visual stimuli^71^. As a highly salient stimulus, angry facial expressions have been shown to capture attention to a greater extent^72^ and produce greater neural attenuation compared to neutral stimuli^38,39^. Elevated HEP amplitude is commonly viewed as an enhanced interoceptive response to cardiac signals^14–16^. In our paradigm, this may be achieved by interoceptive learning via the encounter of similar heartbeat patterns in repeating trials. Based on this interpretation, HEP reduction to repeated negative faces may reflect an attentional weighting process between exteroceptive and interoceptive signals which impacts on interoceptive learning for this particular stimulus category. A possible interpretation would be that angry faces may receive more attention due to their highly salient nature, shifting focus from the interoceptive to the exteroceptive domain. This mechanism may be exacerbated by the presence of an explicit cue enabling the formation of strong expectations about an upcoming negative stimulus in the exteroceptive domain. Our data pattern in response to repeating angry facial expression may thus reflect the relative confidence in descending predictions and ascending prediction errors^73^, which produces a strong repetition suppression effect in the exteroceptive domain but impacts on the learning mechanism in the interoceptive domain indexed by repetition enhancement of the HEP, thereby giving rise to the significant correlation observed between the HEP and VEP in this particular stimulus category. The idea of an attentional weighting process to explain our data pattern highlights that while our cue-based effect emphasises the contribution of top-down mechanisms to the observed data pattern, we do not necessarily attribute our findings purely to these. For example, the mismatch negativity literature which uses similar designs to manipulate the predictability of stimulus sequences, contains several alternate mechanistic accounts to explain a change of neural activity to unpredictable oddballs^74–76^. While interpretations based on predictive-coding unify many competing theories to provide a more parsimonious account of the mismatch negativity^30^, it is generally assumed that this phenomenon is the result of a combination of prediction error and involuntary attentional processes such as attention switching^77^ or contrast enhancement^78^. The interplay between attention and predictive coding was likewise highlighted by Schröger, Marzecová & SanMiguel^79^ within the auditory domain who postulate that attention may function as a gain modulation, enhancing the bottom-up prediction error signal sent to higher levels of the hierarchy. Similar to this literature, we thus do not rule out that our findings may be produced by an interplay of top-down- and attention-based mechanisms which govern the relationship between external and internal perception.

The link between processing extero- and interoceptive signals is further highlighted by the significant correlation between the reduction of VEP and HEP amplitude. The relationship between both components implied by this result highlights that the expression of both components may be influenced by the same governing mechanism which could reflect the allocation of attentional resources across intero- and exteroceptive domains in the face of highly salient, potentially threatening information. The significant correlation between neural measures of external and internal processing also emphasises one of the major findings of our study. Across both experiments, we discovered that the perception of exteroceptive visual information is able to modulate the expression of interoceptive markers. To date, work has shown the reverse, reporting that interoceptive signaling can have a significant impact on the perception of exteroceptive stimulus material. For example, Allan and colleagues^80^ reported that cardiac acceleration to unexpected, arousing stimuli modulates perceptual confidence. Similarly, Park and colleagues^13^ demonstrated that higher HEP amplitude measured in the right inferior parietal lobule and the ventral anterior cingulate cortex predicted improved detection of a faint visual grating stimulus. To our knowledge, our findings are the first to extend this literature by reporting that the perception of exteroceptive stimulus material can likewise impact on the signaling of interoceptive cardiac commands.

Finally, we wish to highlight several limitations relating to the presented work and highlight potential ways in which its contributions could be extended. One of our study limitations is that we did not obtain respiratory parameters in our current paradigm and are thus unable to account for their potential impact on our findings concerning the HEP. As the HEP can be affected by several cardiorespiratory parameters, future work would benefit from including these measures. Relatedly, the short inter-trial interval of our experimental set-up did not allow measurement of cardiac parameters such as the interbeat interval and activity in the heart period power spectrum during the experimental recording^81^. Thus, while we tested whether general variation in these parameters among our participants affected HEP expression by exploring their association during the heartbeat tracking recording, we were unable to test for any association between HEP expression and heartrate variability in the experimental recording. Importantly, we could rule out that general fluctuations of cardiac activity within our participant sample affected HEP expression. We tested for this by regressing both the interbeat interval and high/low heart period power on HEP amplitude during the heartrate tracking interval. Our findings revealed no impact of either of these variables, thus highlighting that HEP amplitude remained unaffected by potentially varying cardiac parameters within our participant sample. Given the potential contribution of attentional mechanisms to our data pattern for negative repeated faces, a worthwhile extension of our study would lie in pairing the existing paradigm with an attentional manipulation to contrast conditions in which stimuli are freely attended with conditions in which attention is diverted. This would explore whether attention mediates the relationship between intero- and exteroceptive sensory signaling. Similarly, it would be interesting to explore whether altered modulation of neural signatures is exclusive to stimuli of negative valence or whether higher emotional salience in general produces the data pattern we observed for angry facial expressions. Introducing a further set of positive facial expressions in addition to neutral and negative ones would answer this question and provide further insight into this phenomenon.

In conclusion, findings of the current dataset are threefold. Firstly, we demonstrate effects of stimulus repetition for evoked potentials in the interoceptive as well as the exteroceptive domain, a novel phenomenon in the literature dealing with repetition suppression and repetition enhancement. Secondly, our cue-based manipulation provides further evidence for a top-down account of repetition suppression for early visual evoked components. Our dataset hereby strengthens theories conceptualising neural attenuation as a function of minimised prediction error achieved by updating a perceptual model at higher levels of the sensory hierarchy. Furthermore, we provide a potential indication of this perceptual learning mechanism for interoceptive cardiac signals in the form of HEP repetition enhancement. Finally, our findings provide evidence that the perception of exteroceptive stimuli can modulate interoceptive neural markers, thus strengthening the association between extero- and interoceptive perception highlighted by previous work. Combined, our work emphasises the importance of considering the impact of top-down mechanisms underlying associative learning, such as sensory prediction and minimisation of prediction error for the processing of extero- and interoceptive signals and highlights the necessity of exploring interoceptive signaling in light of exteroceptive influences. Considering the generation of interoceptive states as a dynamic system which interfaces with incoming environmental information will lead to a more ecologically valid approximation of the phenomenon and an advanced understanding of how interoception and associated states such as embodied selfhood are generated.
